# Cellular Transplantation Alters the Disease Progression in Becker's Muscular Dystrophy

**DOI:** 10.1155/2013/909328

**Published:** 2013-06-06

**Authors:** Alok Sharma, Amruta Paranjape, Hemangi Sane, Khushboo Bhagawanani, Nandini Gokulchandran, Prerna Badhe

**Affiliations:** ^1^Department of Medical Services and Clinical Research, NeuroGen, Brain and Spine Institute Private Limited, Surana Sethia-Hospital and Research Centre, Suman Nagar, Sion-Trombay Road, Chembur, Mumbai, Maharashtra 400071, India; ^2^Department of Neuro-Rehabilitation, NeuroGen, Brain and Spine Institute Private Limited, Surana Sethia-Hospital and Research Centre, Suman Nagar, Sion-Trombay Road, Chembur, Mumbai, Maharashtra 400071, India; ^3^Department of Research and Development, NeuroGen, Brain and Spine Institute Private Limited, Surana Sethia-Hospital and Research Centre, Suman Nagar, Sion-Trombay Road, Chembur, Mumbai, Maharashtra 400071, India; ^4^NeuroGen, Brain and Spine Institute Private Limited, Surana Sethia-Hospital and Research Centre, Suman Nagar, Sion-Trombay Road, Chembur, Mumbai, Maharashtra 400071, India

## Abstract

Becker's Muscular Dystrophy (BMD) is a dystrophinopathy manifested as progressive muscle degeneration. Autologous Bone Marrow Mononuclear Cells (BMMNCs) have shown some myogenic potential. The paracrine effects of the BMMNCs reduce the inflammation and are thought to reduce muscle degeneration. We treated a 39 year old dental surgeon suffering from BMD. Muscle strength was reduced when measured using modified Medical Research Council's Manual Muscle Testing (mMRC-MMT). Static sitting balance was poor. He was wheelchair dependent for ambulation and moderately independent in Activities of Daily Living (ADL). Functional Independence Measure (FIM) score was 93. Musculoskeletal Magnetic Resonance Imaging (MRI-MSK) showed moderate fatty infiltration in the muscles. Three cellular transplantations were carried out. Clinical assessment and the investigations were repeated. Progressive increase in the muscle strength was noted. Ambulation was independent using push-knee splints and minimal assistance when weary. Static and dynamic balance in sitting and standing improved. FIM score increased from 93 to 105. There was no increase in the degree of fatty infiltration, as seen on the MRI-MSK. The case study provides evidence for the putative benefits of cellular therapy in altering the disease progression in BMD. It also suggests augmented clinical benefits of combination of cellular therapy and rehabilitation.

## 1. Introduction

Becker's Muscular Dystrophy (BMD) is one of the dystrophinopathies caused due to in-frame deletions of the exons of dystrophin gene leading to incomplete translation of its protein product, Dystrophin [1]. This incomplete translation leads to functionally incompetent protein [2]. Dystrophin is essential to maintain the structural integrity of the muscle fibers against the mechanical and contractile stresses [3]. In absence of dystrophin, there is increased breakdown of muscle fibers and increased phagocytosis. In the early phase of the disease, this is compensated by regeneration of new muscle fibers from quiescent satellite cells. However, limited numbers of satellite cells leave the rampant muscle necrosis uncompensated as the disease progresses [4]. Clinically, this is manifested as progressive muscle weakness and wasting leading to loss of functionality. There is a vast variation in the clinical manifestation of this disease [5]. BMD leads to severe loss of function and disability in most part of the life followed by premature death [6].

Management of BMD consists of use of corticosteroids to reduce the inflammatory breakdown of the muscle fibers and delaying the progression of the disease. It also includes medical management of the fatal manifestations of cardiomyopathy and multidisciplinary rehabilitation [6, 7]. So far, the management of BMD is only aimed at maintaining the highest possible functionality in an individual, however, the impending fate of the disease cannot be altered. The logical cure of the disease lies in correcting the genetic defect. Although some attempts at gene therapy have been made, swift clinical success of gene therapy seems distant [8]. Cellular therapy has shown some promise in being able to regenerate muscle fibers and regain dystrophin expression after the transplantation of the precursor cells [9–11]. We present our findings in a case of BMD treated with autologous bone marrow mononuclear cells (BMMNCs) followed by rehabilitation and monitored over a period of 24 months.

## 2. Case Report

A 39-year-old dental surgeon visited our center. He was easily fatigable as a child and suffered frequent falls while running. At the age of 12, the symptoms became more evident with difficulty in climbing stairs. As the weakness in lower limbs progressed, he sought medical advice. Based on the clinical features and electromyogram and nerve conduction velocity (EMG-NCV) findings, he was diagnosed with Becker's muscular dystrophy at the age of 15 years. In the third decade of his life, he experienced diffuse myalgia and difficulty in overhead activities due to weakness. He was wheelchair bound by the age of 34.

He was assessed thoroughly when he visited our center. We confirmed the diagnosis with multiplex polymerase chain reaction (PCR) testing for 32 exons which revealed in-frame deletion of exons 45, 46, and 47 [13]. Neurologically he presented with hypotonia and diminished reflexes. Hip muscle tightness of right hip flexors and right iliotibial band was observed on examination. Muscle strength was assessed using modified Medical Research Council's manual muscle testing scale (mMRC-MMT) (Table 1). This grading was designed to be able to detect the smaller changes in the muscle strength than assessed by the Medical Research Council (MRC) grading. The details of the muscle strength charting of all the muscles is given in Table 2. His static balance in sitting was poor. He was unable to stand with or without support. For assessing the degree of independence in the activities of daily living (ADL), Functional Independence Measure (FIM) scale was used and the score was 93. He was completely dependent for transfers from wheelchair to bed, bed to wheelchair, wheel to toilet, and toilet to wheelchair. He needed help to set up the equipment at his workplace. With extreme difficulty, he could continue working as a dentist as the shoulder and wrist strength was only of functional grade. EMG-NCV and Magnetic Resonance Imaging-Musculoskeletal (MRI-MSK) findings were consistent with the diagnosis. MRI-MSK revealed extensive fatty infiltration in all the muscles of the hip and moderate fatty infiltration in the anterior, lateral, and posterior compartment muscles of the leg.

## 3. Intervention

A duly filled informed consent was obtained. Selection of this patient for the treatment was based on World Medical Association's Revised Declaration of Helsinki [14]. The ethical approval was obtained from Institutional committee for Stem Cell Research and Therapy (IC-SCRT), NeuroGen, Brain and Spine Institute, Mumbai, India.

Preoperative fitness was assessed by serological, biochemical, and hematological blood tests, chest X-ray, electrocardiogram (ECG), and 2D Echocardiography testing a week before adult autologous BMMNCs transplantation. Enhanced mobilization of the cells was ensured by administering Granulocyte Colony Stimulating Factor (GCSF) subcutaneously 48 hours and 24 hours prior to the MNCs transplantation [15]. On admission, detailed assessment was carried out by a range of medical and allied health professionals. To ensure the cell fraction received by each muscle is significant, only the muscles with mMRC-MMT score less than 3 and of functional importance were selected. Motor points, the point where the innervating nerve enters the muscle belly, of these muscles were identified and marked by an experienced physiotherapist. On the day of transplantation, 100 mL bone marrow was aspirated from anterior superior iliac spine under local anesthesia, using bone marrow aspiration needle and was collected in heparinized tubes. Mononuclear cells (MNCs) were separated using density gradient method. Fluorescence activated cell sorting (FACS) analysis showed 94% viability of the cells and CD34+ count to be 1.06%. Half of the cells were injected intrathecally at the level between L4 and L5. The remaining cells were then diluted in cerebrospinal fluid (CSF) due to the properties of CSF that harbors cell growth [16]. There were then injected intramuscularly, bilaterally in the specific motor points of Biceps, Triceps, Hamstrings, Quadriceps, Glutei, Back extensors and Abdominals. Methyl prednisolone 1 gm in 500 mL of Ringer Lactate solution was administered intravenous simultaneously to decrease the immediate inflammation. The total number of cells injected, via both the routes combined was 56 × 10^6^.

This was followed by multidisciplinary rehabilitation. Physiotherapy consisted of bed mobility exercises, training for various transfers, and suspension exercises for the muscles with mMRC-MMT grade below 3. He was trained for standing with push-knee splints and high boots with steel shanks. Exercises aimed at strengthening the muscles were performed at moderate intensity within the fatigue range. Occupational therapy consisted of strengthening exercises of bilateral upper limb and trunk muscles and training for ADL. Counseling was provided by a psychologist to cope better with the disease.

The patient was discharged at one week and was advised to continue the rehabilitation at home under the guidance of rehabilitation professional. A detailed follow-up assessment was conducted after three and seven months. At seven months, MRI-MSK scan and EMG-NCV study were repeated. MRI-MSK was conducted on the same MRI unit with the same parameters as earlier. In the view of the positive response to the treatment (as explained in results), patient underwent second cellular transplantation seven months after the first transplantation. The clinical reasoning behind the subsequent transplantation is discussed in detail in the discussion section. The transplantation procedure was replicated except for the muscles chosen. Intramuscular injections were given in bilateral rhomboids, deltoid, biceps, triceps, brachioradialis, abdominals, back extensors, glutei, quadriceps, hamstrings, and hip adductors. Eight months after the second transplantation, patient underwent third transplantation. Procedure was identical and the muscles chosen for intramuscular injections were quadriceps, glutei, hamstrings, deltoid, biceps, triceps, brachioradialis, rhomboids, and abdominal and back extensors. 

## 4. Results

At one week post cellular transplantation, there was a flicker of contraction noted in the Biceps bilaterally. Exercise tolerance had increased. The diffuse myalgia had reduced. He could walk using calipers and minimum assistance from the therapist.

Three months after transplantation, the myalgia resolved completely. There was a palpable contraction of the biceps bilaterally, and muscle strength had improved from grade 0 to grade 1 according to mMRC-MMT grading. With some difficulty and occasional assistance for fastening the belt and buttoning, he was independent in wearing his pants. Sitting without support was possible. Standing without support wearing the push knee splints and high boots, was easier with improved static standing balance. He could also take a few steps.

Seven months after the first transplantation, his standing balance had improved; further, he successfully performed multidirectional reach outs. Transfer from his chair to bed and from bed back to his chair was independent. Rolling in bed was possible without any assistance. He could dress up faster now and was completely independent in upper and lower body dressing. The muscle strength as measured by modified mMRC-MMT showed an increase from grade 0 to grade 1 in biceps, brachialis, brachioradialis, triceps, quadriceps, and internal rotators of the hip. Walking was possible with maximal support and push-knee splints, once in a day limited to 15–20 minutes due to high fatigability. MRI and EMG-NCV showed no increase in the dystrophic changes of the muscles, suggesting maintained muscle integrity.

Fifteen months after the first transplantation and eight months after the second transplantation, there was significant improvement in the dynamic balance while standing and sitting. He started walking wearing the splints, without any assistance and only minimal assistance when fatigued, for up to half an hour twice a day. He successfully climbed 5-6 stairs with support of the railing. While performing his vocational activities the trunk control was better. He reported increased exercise tolerance and could perform the exercises faster and with greater ease. The time taken to complete the exercise routine had reduced by one hour. Upper and lower body dressing, transfers, and toileting activities could be performed with no assistance. FIM score increased from 93 to 105. Upper extremity strength was reported to be maintained with no further difficulty in carrying out the vocational activities in fifteen months. As noted by the treating therapist, quality of the movement was better, and there was no need to stabilize the hip while walking. Various muscle groups had gained strength as shown in Table 3 and strength gains achieved after the previous transplantation were maintained.

The improvement was maintained for 2 years after the first transplantation.

## 5. Discussion

Stem cells can be defined as cells that give rise to a committed progeny of a specific tissue type or self-renew as the clonal precursors or differentiate into the precursor cells belonging to germ layers other than that of the original cell [17]. Adult human tissues undergo continuous self-renewal or self-repair. Quiescent stem cells therefore exist in abundance. Their regeneration potential varies depending on the body systems, and hematopoietic cells undergo continuous renewal into multiple blood cell types and are a rich source of stem cells, just like skin epithelial cells and adipose tissue [18–20]. Nervous tissue also shows presence of some stem cells; however, evidence regarding their regeneration potential remains less understood [21]. Skeletal muscles also exhibit tremendous potential to regenerate and repair following an injury owing to the tissue specific stem cells, satellite cells. Skeletal muscles consist of myofibers which are multinucleate syncytial cells, highly specialized in their structure for their contractile properties [22]. Satellite cells are situated under the basal lamina of the myofibers. These cells show tremendous potential to generate terminally differentiated myocytes [23] or self-renew to maintain the pool of quiescent cells for future demands [24]. There are presumed to be subpopulations of satellite cells. One subset which can undergo rapid terminal differentiation but shows only limited potential to regenerate, and the other is in primitive stages of differentiation, which slowly regenerates large number of muscle cells [25]. In a disease like muscular dystrophy where there is a progressive disintegration of muscle tissue, both these populations are exhausted rapidly. Although satellite cell transplantation seems like an enticing option for treating muscular dystrophy, satellite cells lose their regeneration potential when isolated by conventional techniques. Newer techniques are being devised but are distant [26]. The obvious subsequent choice is the muscle precursors, myoblasts. Myoblasts, however, have limited migration and regeneration potential when transplanted intramuscularly; they cannot be delivered via any other route, are difficult to isolate (isolation requires muscle biopsy), and therefore have been unsuccessfully used to treat muscular dystrophies in experimental settings [27–29].

When thought about the characteristics of ideal stem cell to treat muscular dystrophy one may agree with Meng et al., 2011. Ideally, the stem cell should have the capacity to expand in vitro with high migration and regeneration potential and easy systemic delivery. They should also be myogenic, able to reconstitute the satellite pool, and regain the dystrophin expression. Although some cell types may qualify as ideal stem cells, isolating and transplanting them are only hypothetical at this juncture [30]. There is some evidence suggesting myogenic potential of autologous BMMNCs [31, 32]. Being separated from the hematopoietic cell population, they are available in abundance and can be isolated easily and delivered systemically. Some preclinical studies have also shown dystrophin expression following BMMNCs transplantation. Although the number of muscle fibers regenerated and their functional potential is debated [31, 32], BMMNCs benefit muscles in the necrotic environment by reducing the inflammation [33], facilitating angiogenesis [34], secreting various growth factors [35], monitoring cell apoptosis [36], and tempering the immune system [37]. Autologous BMMNCs was therefore the choice of transplantation in this case.

Pathological process of muscular dystrophy mostly affects the muscles; however, there is some evidence of involvement of neural structures [38]. BMD also manifests as cognitive and psychological affectation [39]. Dystrophin is believed to play some role in neuronal synapses. There is some evidence of presence of dystrophin in nicotinic synapses and its role in cellular interactions. It is also believed that it helps to maintain the integrity of myelin sheath by better anchoring the schwann cells [40]. Cotransplantation of myoblasts in presence of schwann cells has shown greater therapeutic potential in preclinical studies [41]. These findings identified the need to introduce BMMNCs in the neural system. Intravenous administration would involve a high risk of differentiation into other cell types and migration to various organs; therefore, we chose a more intimate environment through intrathecal and intramuscular transplantation. Muscular dystrophy is a progressive condition, and a single transplantation may not be sufficient to reconstitute the depleted satellite cell pool; therefore, subsequent transplantations were carried out. Rehabilitation following stem cell transplantation has been shown to have greater therapeutic effect [42]. Physical training and endurance training is beneficial in maintaining the cardiac status and functional level in BMD [43, 44]. In our case study, we observed augmentative benefits of rehabilitation combined with cellular therapy.

Increase in FIM scores have previously been shown to have modest correlation with quality of life in various neuromuscular conditions [45]. We have also found similar clinical benefits in patients suffering from Duchenne Muscular Dystrophy (DMD) earlier [46]. We have used MRI-MSK earlier to assess the effects of cellular therapy, suggesting regeneration of muscle fibers post cellular therapy in DMD [47]. BMD is associated with progressive increase in fatty infiltration of muscle tissue. In this case study, the comparison of MRI findings before cellular therapy with 8 months and 15 months post cellular therapy showed no increase in the fatty infiltration. These MRI findings and repeated clinical assessments further substantiate the putative benefits of cellular therapy in altering the disease progression. BMD also causes linear regression of strength. Increase in palpable and clinically measurable muscle strength in this case suggests regeneration of some functional muscle fibers. It is also interesting to note that the muscles that have regained strength were the ones that were chosen for either two or all three transplantations. Frequent cellular transplantation may compensate, to an extent, the deficit between muscle necrosis and regeneration. It may also prevent the fibrotic replacement of the muscle tissue and therefore help alter the progression of the disease.

## 6. Conclusion

The case study provides evidence for the putative benefits of cellular therapy in altering the disease progression in BMD. It also suggests augmented clinical benefits of combination of cellular therapy and rehabilitation. Although it is one of the initial cases to report tangible changes on clinical examination, a singular case report is too early to draw any conclusions. The possibilities of using various cell types with different protocols must be tested with rigorous research protocols to ascertain the effectiveness of cellular therapy for the treatment of BMD.

## Figures and Tables

**Table 1:** Modified medical research council manual muscle testing scale. Florence et al, 1992 [12].

mMRC-MMT scale grades	Description
0	No Movement
1	A flicker of movement is seen or felt in the muscle
2	Muscle moves the joint when gravity is eliminated
3−	Muscle moves the joint against gravity but not through full mechanical range of motion
3	Muscle cannot hold the joint against resistance but moved the joint fully against gravity
3+	Muscle moves the joint fully against gravity and is capable of transient resistance but collapses abruptly
4−	The same as grade 4, but muscle holds the joint only against minimal resistance
4	Muscle holds the joint against a combination of gravity and moderate resistance
4+	The same as grade 4, but muscle holds the joints against moderate to maximal resistance
5−	Barely detectable weakness
5	Normal strength

**Table 2:** mMRC-MMT scale grading for all the muscles as examined before stem cell transplantation.

Muscle group tested	mMRC-MMT score on the right side	mMRC-MMT score on the left side
Hip		
Flexors	2	2
Extensors	1	1
Abductors	2	2
Adductors	1	1
Internal rotators	0	0
External rotators	0	0
Knee		
Flexors extensors	2	2
	0	0
Ankle		
Dorsiflexors	3	3
Plantar flexors	3+	3+
Invertors	3	3
Evertors	3	3
Shoulder		
Flexors	3+	3+
Extensors	3−	3−
Abductors	3+	3+
Adductors	2	2
Internal rotators	3+	3+
External rotators	3+	3+
Elbow		
Biceps	0	0
Brachialis	0	0
Brachioradialis	0	0
Elbow extensors	0	0
Wrist		
Flexors	3	3
Extensors	3	3
Fingers		
Lumbricals	3	3
Palmar interossei	3	3
Dorsal interossi	3+	3+
Trunk		
Upper abdominals	2+
Lower abdominal	1
Back extensors	1

**Table 3:** Changes in the muscle strength over fifteen months after the first cellular transplantation.

Muscle group tested	mMRC-MMT bilaterally before cellular therapy	mMRC-MMT bilaterally 3 months after the cellular therapy	mMRC-MMT bilaterally 8 months after the cellular therapy	mMRC-MMT bilaterally 15 months after the first cellular therapy
Hip flexors	2	2	2	2
Hip extensors	1	1	1	2
Hip abductors	2	2	2	2
Hip adductors	1	1	1	2
Hip internal rotators	0	0	1	1
Hip external rotators	0	0	0	1
Knee flexors	2	2	2	2
Knee extensors	0	0	1	2
Tibialis anterior	3	3	3	3+
Tibialis posterior	3	3	3	3+
Extensor halluces longus	3	3	3	3+
Extensor digitorum	3	3	3+	3+
Shoulder extensors	3−	3−	3	3+
Biceps	0	1	1	1
Brachialis	0	0	1	1
Brachioradialis	0	0	1	1
Triceps	0	0	1	1
Supinators	2	2	2	3−
Wrist extensors	3	3	3	3+
Palmar and dorsal interossi	3	3	3	3+
Lumbricals	3	3	3	3+
